# Diagnostic Work-up and Follow-up in Children with Tall Stature: A Simplified Algorithm for Clinical Practice

**DOI:** 10.4274/jcrpe.2220

**Published:** 2015-12-03

**Authors:** Susanne E. Stalman, Anke Pons, Jan M. Wit, Gerdine A. Kamp, Frans B. Plötz

**Affiliations:** 1 Tergooi Hospitals, Department of Pediatrics, Blaricum, The Netherlands; 2 Academic Medical Center, Department of Pediatrics, Amsterdam, The Netherlands; 3 Leiden University Medical Center, Department of Pediatrics, Leiden, The Netherlands

**Keywords:** children, growth monitoring, height, target height, tall stature, overgrowth, height reduction

## Abstract

**Objective:**

No evidence-based guideline has been published about optimal referral criteria and diagnostic work-up for tall stature in children. The aim of our study was to describe auxological and clinical characteristics of a cohort of children referred for tall stature, to identify potential candidates for adult height reduction, and to use these observations for developing a simple algorithm for diagnostic work-up and follow-up in clinical practice.

**Methods:**

Data regarding family and medical history, auxological measurements, bone age development, physical examination, additional diagnostic work-up, and final diagnosis were collected from all children referred for tall stature, irrespective of their actual height standard deviation score (HSDS). Predicted adult height (PAH) was calculated in children above 10 years. Characteristics of patients with an indication for adult height reduction were determined.

**Results:**

Hundred thirty-two children (43 boys) with a mean ± SD age of 10.9±3.2 (range 0.5-16.9) years were included in the study. Fifty percent of the referred children had an HSDS ≤2.0 (n=66). Two pathological cases (1.5%) were found (HSDS 2.3 and 0.9). Tall children without pathology were diagnosed as idiopathic tall, further classified as familial tall stature (80%), constitutional advancement of growth (5%), or unexplained non-familial tall stature (15%). Of the 74 children in whom PAH was calculated, epiphysiodesis was considered in six (8%) and performed in four (5%) patients.

**Conclusion:**

The incidence of pathology was very low in children referred for tall stature, and few children were potential candidates for adult height reduction. We propose a simple diagnostic algorithm for clinical practice.

**WHAT IS ALREADY KNOWN ON THIS TOPIC?**

Various expert-based flow charts about diagnostic work-up for tall stature have been proposed, focusing on identifying pathological causes for tall stature. No exact data are available on the incidence of pathological causes of tall stature, but most clinicians consider these rare.

**WHAT THIS STUDY ADDS?**

Our study shows an incidence of pathological causes of perceived tall stature of 1.5% and that adult height reduction is seldom indicated. Of the non-pathological cases, most are familial (compared with sex-corrected midparental height as well as with height of the tallest parent), but constitutional advancement of growth and unexplained non-familial tall stature are also observed. We suggest a simplified diagnostic algorithm and recommendations for follow-up.

## INTRODUCTION

Tall stature is defined as a height of more than 2.0 standard deviations (SD) above the corresponding mean height for age and sex as observed in the population (1,2). Although there are as many children with tall stature as children with short stature, tall stature is a less common reason for referral from primary health care to specialist care than short stature (2).

There are two main reasons to refer children with tall stature. First, like in short stature, it is important to distinguish between normal variation and pathology (3). There is some confusion about the nomenclature of non-pathological causes of tall stature. In the European Society for Paediatric Endocrinology (ESPE) Classification of Paediatric Endocrine Diagnoses (3), this group was denominated as idiopathic tall stature (ITS) and further subdivided into genetic (familial) tall stature (or constitutional tall stature) and non-familial tall stature (NFTS). Later, several authors coined the term “constitutional advancement of growth (CAG)” (1,3,4) for tall children with a height beyond the target height (TH) range but with coincident bone age (BA) advance, who therefore would be expected to end up with a normal adult height. The second reason for referral of tall children is to predict adult height and thus to identify potential candidates for adult height reduction (5). Recommendations regarding follow-up of tall children are lacking, in particular with respect to indications for interventions to reduce adult height. We have learned from clinical experience that some children with a normal height can reach very tall adult stature if bone maturation and/or puberty are extremely delayed. In addition, only few data have been published about the characteristics of patients who eventually underwent adult height reduction by epiphysiodesis (5,6).

So far, no evidence-based (inter) national guideline has been published about optimal referral criteria, diagnostic work-up, and follow-up for tall stature. Various expert-based algorithms have been proposed to identify pathological causes although in the majority, no pathology can be found (1,4,7). Here, our first aim was to describe auxological and clinical characteristics of a cohort of children referred for tall stature to a general pediatric clinic. Our second aim was to identify potential candidates for adult height reduction. Based on these observations and published literature, we propose a simple algorithm for diagnostic work-up and follow-up of children with tall stature which, we hope, will be prospectively validated in the future.

## METHODS

### Study Population

In this study, we included all children referred for tall stature, irrespective of their actual height standard deviation score (HSDS), to the general (non-academic) pediatric growth clinic of Tergooi Hospitals in the Netherlands between January 2010 and March 2014. Exclusion criteria were ethnicity other than Dutch (defined as at least one parent with non-western European ethnicity) and missing medical records.

### Data Collection

All patients underwent a four-step diagnostic program. First, the parents completed a questionnaire regarding perinatal and medical history (with special attention to psychomotor/mental development and behavior), medication use, pubertal signs, growth and family history regarding height, puberty onset and medical history of first-degree relatives. Second, BA was assessed by a single pediatrician (G.K.) according to the method of Greulich and Pyle (8) and BA advance or delay was calculated as BA minus chronological age (CA). Third, height, weight, head circumference, sitting height (SH), arm span, blood pressure, and heart rate were measured. Parental height could be measured in most cases (approximately 95%), the remaining parental height data were based on reported values. TH was calculated based on father’s height (FH) and mother’s height (MH) according to two equations, both without secular trend correction. We used the traditional Tanner formula [(FH+MH)/2+ or -6.5] (9) and conditional TH (cTH) based on the most recent Dutch growth study reported by van Dommelen et al (10) (44.5+0.376xFH+0.411xMH for boys and 47.1+0.334xFH+0.364xMH for girls). SDS of the following items were calculated based on appropriate reference data: height (HSDS) (11); TH (in cm and SDS) (9) and cTH (in cm and SDS) (10); body mass index (BMI) (12); head circumference (13); sitting height/height ratio (SH/H) (14); and birth weight, length and head circumference (15). The HSDS distance to THSDS according to Tanner as well as to cTHSDS according to van Dommelen et al (10) was calculated, and we also calculated the difference between the patient’s HSDS and the HSDS of the tallest parent. Predicted adult height (PAH) was calculated in children above 10 years according to Bayley and Pinneau (16) and an update of the formula of De Waal et al (17) based on the change of the TH formula because of the discontinuation of secular trend in the Netherlands (11): 267.02+(0.62xheight) + (2.75xcTHSDS) - (10.49xCA) - (12.98xBA) + (0.72xCAxBA) for boys and 158.42+(0.74xheight) + (1.47xcTHSDS) - (5.90xCA) - (7.70xBA) + (0.41xCAxBA) for girls (Wit and van Dommelen, personal communication). Finally, one pediatrician (G.K.) reviewed the questionnaire, BA, and the auxological measurements and performed a complete physical and neurological examination. The physical examination was focused on detecting body disproportion (defined as SH/H ratio >-2.2 SDS) (14) and specific dysmorphic features rated on syndrome-specific checklists. Pubertal development was rated according to Tanner (18,19).

### Diagnostic Work-up

Briefly, diagnostic work-up in order to uncover pathologic causes for tall stature was considered in case of presence of the following features: developmental and/or speech delay, behavioral problems, dysmorphisms, disproportion, a distance between HSDS and THSDS >2.0, a recent growth acceleration, pubertal development not appropriate for age, or indications of other hormonal disorders such as growth hormone excess or hyperthyroidism.

### Definitions

Pathological tall stature was subclassified into two categories: children with a dysmorphic syndrome with overgrowth (primary growth disorder) and those with tall stature caused by endocrine diseases (secondary growth disorder) (3). Non-pathological tall stature is considered a normal variant, probably caused by multiple gene variants with a positive effect on linear growth (20) and maturation. Because the precise etiology is unknown, this condition is termed ITS in the ESPE Classification of Paediatric Endocrine Diagnoses (3), and further classified into two subclasses: familial tall stature (FTS) (explained by gene variants associated with linear growth) and NFTS. In our study, we further distinguished in the latter group two conditions: CAG (hypothetically explained by gene variants associated with a fast tempo of growth, “maturation”) and others unexplained by genetic factors.

Three ways to distinguish FTS from were used. First, FTS was defined as a HSDS distance to THSDS <2.0 based on the Tanner equation and TH range of +/- 2.0 SD (9). Second, we subclassified the children based on the distance to cTHSDS (10), with a cTH range of +/-1.6 SD (children with a HSDS minus cTHSDS <1.6 were classified as FTS). Third, we reasoned that tall height can appear as a dominantly inherited condition, either monogenic (e.g., in Marfan syndrome), or polygenic (if the child inherits by chance mostly “tall gene variants” from the tallest parent). So, tall children were also denominated as FTS if they were tall (HSDS>+2.0) and their HSDS minus HSDS of the tallest parent was below +1.6. CAG was defined as NFTS with an advanced BA >2.0 years (8).

Children with a HSDS ≤2.0 were classified as ‘not-tall’. These children were included in the study because the reason for referral was identical to that of children with a HSDS >2.0. Characteristics of not-tall and tall children were compared, including their HSDS, THSDS, HSDS-THSDS, BMI, BA-CA, and PAH data.

### Adult Height Reduction

Adult height reduction by epiphysiodesis was considered for a PAH of >205 cm (+3.0 SDS) in boys or >185 cm (+2.3 SDS) in girls (5) with PAH based on the formula of De Waal et al (17), since this is considered the most accurate for tall stature in the Netherlands. These patients were assessed separately to determine the characteristics of this group. Furthermore, children with a PAH >205 cm (boys) or >185 cm (girls), regardless of their HSDS at presentation, were assessed to identify patients with a HSDS ≤2.0 at presentation who might still have an indication for adult height reduction and to determine their characteristics.

### Analysis

All data were collected from hospital files and were analysed in Statistical Package for the Social Sciences, version 19. Descriptive statistics were used to quantify the prevalence of pathologic causes for tall stature and the analysis of the different diagnostic group characteristics. Independent t-tests for continuous variables and chi-square tests for categorical variables were used to compare characteristics between boys and girls and between different diagnostic groups. A value of p<0.05 (two-sided) was considered statistically significant.

### Ethical Approval

Approval for the study was obtained by the Scientific Review Committee of Tergooi Hospitals (letter reference kv/15.05).

## RESULTS

### Study Population

During this study period, 139 children were referred for tall stature, of whom seven children were excluded because of missing data (n=4) or non-Dutch ethnicity (n=3). Patient characteristics of the 132 children included for analysis are shown in Table 1.

### Diagnostic Work-up

The adherence to our initial four-step diagnostic work-up protocol was 100% and additional diagnostic work-up was performed in 19 patients (14.4%) based on clinical judgement using a local protocol and various flow charts (1,4,7). In 5 children with dysmorphic features, one of whom showed slight body disproportion (long legs), genetic testing revealed no specific diagnosis (Marfan syndrome was excluded). In ten children, the head circumference was >2.0 SDS. Given the low Sotos score on the checklist (21), no diagnostic work-up was indicated. Thirteen children did not meet any of the three definitions for FTS (HSDS-THSDS >2.0, HSDS-cTHSDS >1.6, and HSDS-HSDS of tallest parent >1.6). Three of these children had an advanced BA of >2.0 years and were classified as CAG. Seven children had an advanced BA of >1.0 year (1.2-1.9 SDS). Additional work-up was performed in two out of these seven children to rule out secondary growth disorders, which revealed no abnormalities. In the remaining five, no diagnostic work-up was performed, but during follow-up, no abnormal growth pattern was observed. In two out of three pre-pubertal patients with a recent growth acceleration, additional work-up was performed and revealed no specific diagnosis. In six patients with a pubertal development not appropriate for age, the additional work-up showed precocious puberty in two patients. Diagnostic work-up to reveal secondary growth disorders was performed in six children and found negative.

### Diagnoses

A pathological cause of tall stature was found in the two patients with precocious puberty (1.5%). In one patient, the diagnosis was made based on clinical features (recent growth acceleration, HSDS 2.3, an advanced BA development, a reported onset of puberty of 7.5 years, and Tanner pubertal stage B3/P3 at the age of 9.4 years) (22). In the other patient (5.3 years), the diagnosis was based on clinical features (pubertal development, Tanner B2/P1, HSDS 0.9) and a positive gonadotropin-releasing hormone test.

**Figure 1:**
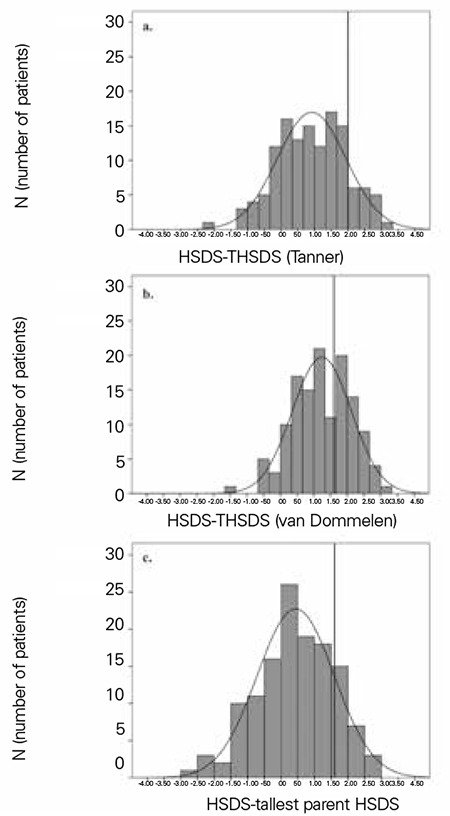
Distributions of HSDS distance to THSDS (a), conditional target height standard deviation score (b) and HSDS distance to tallest parental HSDS (c) HSDS: height standard deviation score, THSDS: target height standard deviation score

**Figure 2:**
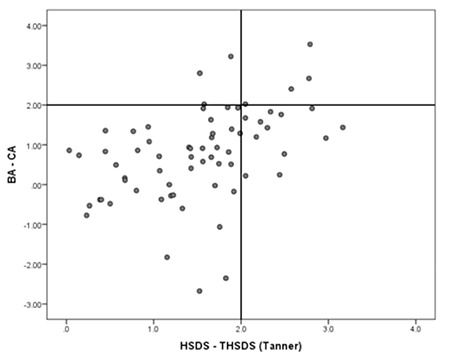
Correlation between HSDS distance to THSDS (according to Tanner) and bone age advancement HSDS: height standard deviation score, THSDS: target height standard deviation score, BA: bone age, CA: chronological age

Figure 1 shows histograms of HSDS minus THSDS (a), HSDS minus cTH (b) and the child’s HSDS minus HSDS of the tallest parent (c). The mean values are 0.9, 1.2, and 0.4, showing that the child’s height is on average closest to the tallest parent’s height. The number of idiopathic tall children labelled FTS according to the three definitions (HSDS-THSDS >2.0, HSDS-cTH >1.6 and HSDS-HSDS of tallest parent >1.6) was 50 (77%), 21 (32%), and 46 (71%), respectively. Fifty-two children complied with at least one of these definitions (80%) and were labelled FTS. Of the 13 children with NFTS, 3 were labelled CAG. Figure 2 demonstrates the correlation between HSDS distance to THSDS (Tanner) and BA development in the 65 children with non-pathological tall stature, showing a relatively advanced BA development in children who are taller than THSDS (Pearson correlation 0.45, p<0.01).

Exactly fifty percent of the children referred for tall stature had an HSDS ≤2.0 (n=66), so were classified as not-tall (Table 2). There was no difference in THSDS between both groups, but tall children had a significantly more advanced BA development compared to not-tall. To answer the question why so many not-tall children were indeed referred with “a fear to become too tall”, we evaluated their family history regarding parental height and parental pubertal development, showing that in 33 out of 66 (50%) not-tall children, at least one parent had a HSDS >2.0 or had a delayed puberty, compared to 29 out of 66 (44%) tall children (p=0.49).

### Adult Height Reduction

In all 74 children above 10 years of age, PAH was calculated (Table 1). PAH based on Bayley and Pinneau (16) resulted in five boys with a prediction >205 cm and 13 girls with a prediction >185 cm. Of these patients, two boys and two girls had a HSDS ≤2.0. Both girls had a delay in puberty. Using the De Waal method, we found that none of the boys had a PAH >205 cm, while six girls had a PAH >185 cm. One of these girls was not tall, but she had pubertal delay. Four patients (3.0%) wished to be referred for adult height reduction by epiphysiodesis (Table 3). One girl who underwent epiphysiodesis did not meet the criteria for treatment at the first presentation, but did so on return two years later.

## DISCUSSION

In our population referred for tall stature, 50% had indeed a height >2.0 SDS. Two patients were diagnosed with precocious puberty (1.5%); one of them had a HSDS of 0.9, but a recent growth acceleration. Of the idiopathic tall children, 80% were diagnosed as FTS, 5% as CAG and 15% remained unexplained. In all children older than 10 years (n=74) PAH was calculated, and in only six (8%), epiphysiodesis was considered and eventually performed in four (5%) patients.

Although the incidence of pathology in children referred for tall stature is very low, it remains important to rule out chromosomal, genetic, or endocrine disorders. A careful history, in particular developmental delay and behavioral problems, may be suggestive for chromosomal disorders, whereas specific findings on examination, like dysmorphisms and macrocephaly, may be suggestive for specific genetic syndromes. The most common genetic causes of tall stature are Klinefelter syndrome, Marfan syndrome, Sotos syndrome, and Beckwith-Wiedemann syndrome (BWS) (23), with an incidence of 1.1-1.7 per 1000 (24), 2-3 per 10.000 (25), 1 per 15.000 (26), and 1 per 13.700 (27) individuals, respectively. Precocious puberty is the most common endocrine cause of accelerated growth. Based on these numbers, it is not unexpected that no primary growth disorders and only two secondary growth disorders have been diagnosed in our population.

To determine whether tall stature is familial or not, the usual approach has been to compare the child’s height with the height of both parents. Conventionally, according to Tanner et al (9), this has been expressed as the midparental height (TH) with a range of +/- 2.0 SD. In theory, however, the cTH (10,28), with additional corrections for parent-parent and parent-offspring correlations, with a range of +/-1.6 would be expected to be superior. Our results suggest that the cTH range (+/-1.6 SD) may be too strict for defining FTS compared to the Tanner TH range (+/-2.0 SD). This is supported by a previous study showing that only 10% of healthy children show a HSDS outside their TH range (defined as +/-1.5 SD) (29) and by estimates that 50-90% of the height variation is accounted for by genetic factors (30).

An alternative approach is to compare the child’s HSDS with the HSDS of the tallest parent. In theory, this would better accommodate genetic influences of dominant inheritance, inheritance of predominantly “tall” gene variants in case of discrepancy between parental heights (1,20,29). However, no experimental data have been collected about the expected range around the difference between the child’s HSDS minus the HSDS of the tallest parent. For the detection of pathological causes of tall stature, this approach appears as not suitable, because one of the most important disorders to diagnose (Marfan syndrome) is transmitted in a dominant fashion.

Most children with non-FTS show an advanced BA and can be labeled CAG. We have shown that their PAH is not different from that of children with FTS, confirming previous observations (1,31). In our opinion, BA assessment remains an important tool in the differential diagnosis of tall stature.

Assessment of height velocity, expressed as a positive change of HSDS, is part of the diagnostic work-up, since increased height velocity may be associated with several rare hormonal causes of tall stature (secondary growth disorders such as precocious puberty, hyperthyroidism and GH excess) (1). We believe that the criterion of a change in HSDS >1.0, over an undefined period of time, may be a too strict rule in children over the age of 10 years (32), since an acceleration in height velocity is a normal phenomenon related to the pubertal stage. Therefore, we propose to first interpret the height velocity (1) in relation to pubertal development prior to performing additional work-up to exclude hormonal pathology. In case of doubt, a follow-up visit in three months’ time to reconfirm normal pubertal growth and development may prevent unnecessary additional tests and costs. Pubertal development should be interpreted in relation to CA. Both precocious puberty and delayed puberty may be the result of underlying pathology. Precocious puberty causes increased height velocity and thus tall stature during childhood but a reduced adult height. In contrast, children with a delayed puberty may present with a normal stature during childhood but may reach a tall adult height (3).

A major reason for referral is to predict adult height and eventually consider adult height reduction. The present attitude of many pediatric endocrinologists is that interventions to reduce adult height are to be discouraged. Treating tall girls with estrogen preparations is considered obsolete by many, because of its association with fertility disorders in adulthood (33,34). Treatment of tall boys with high-dose androgens is rather frequently associated with side effects, and its effect on adult height is limited (35). At present, only epiphysiodesis can be offered, which is associated with few complications in experienced hands (6). Our analysis showed that there is a large difference in numbers of patients with an indication for epiphysiodesis depending on which prediction method is used. Indeed, we demonstrated an overestimation of adult height according to Bayley and Pinneau (16), particularly in boys, compared to De Waal et al (17), as has been shown in previous studies. Therefore, we advise to use the method of De Waal et al (17) to calculate PAH, to avoid unnecessary referrals. If a BA is determined before girls have reached a height of 170 cm and boys 185 cm, there is sufficient time to discuss the pros and cons of the intervention (5,6).

It is noteworthy that out of the four children in whom epiphysiodesis was performed, one patient had a HSDS ≤2.0 at first presentation. We therefore suggest that even in children who are not tall at first presentation, a second referral at height 170 cm before the age of 12.5 years in girls and 185 cm before the age of 14 years in boys for adult height prediction should be considered if a positive family history of delayed puberty is present.

**Figure 3:**
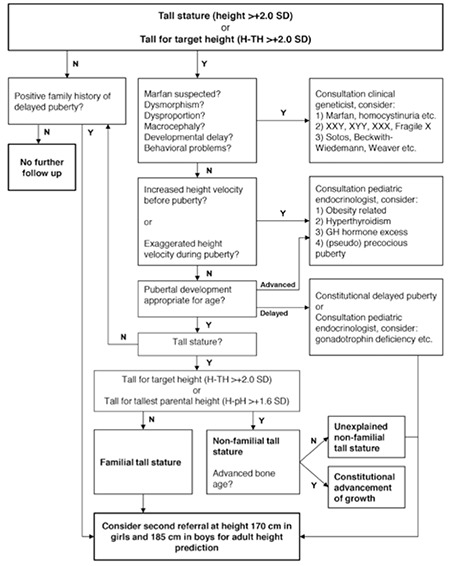
Proposed flow chart 0-17.99 years Proposed flow chart for the diagnostic work-up of tall children aged 0-17.99 years. The flow chart consists of two main pathways. Initially, height standard deviation score and distance to target height (Tanner) are calculated. In case of a height standard deviation score ≤2.0 or height standard deviation score -target height standard deviation score ≤2.0, the child is classified as not-tall or not-tall for target height. We suggest that a second referral at height 170 cm before the age of 12.5 years in girls and 185 cm before the age of 14 years in boys for adult height prediction should be considered if a positive family history of delayed puberty is present. In case of a height standard deviation score >2.0 or height standard deviation score-target height standard deviation score >2.0, each subsequent step is aimed at excluding pathologic causes requiring further investigation and possibly treatment. The final step is to determine whether the tall child is familial tall or non-familial tall

Based on our results and review of the literature, we propose a new diagnostic flow chart (Figure 3). This flow chart uses a simple step by step strategy and suggests recommendations for follow-up. Initially, HSDS and distance to TH are calculated. Each subsequent step is aimed at discovering clues for pathologic causes with severe consequences (e.g. Marfan syndrome) or pathological causes requiring further investigation and possibly treatment (e.g. precocious puberty, hyperthyroidism, and growth hormone excess). The final steps are aimed at classifying children without any established pathology and providing recommendations for follow-up. We suggest a follow-up visit for children with unexplained NFTS and others who may be at risk for attaining an extreme tall stature at a time when they are expected to reach a height of 170 and 185 for girls and boys, respectively.

We acknowledge there are several limitations of this study. First, it is a retrospective study performed in a single centre in a general hospital; it is possible that our study population differs from that in other clinics, particularly academic clinics. Second, our study population contains a limited number of patients, and only few pathologic causes of tall stature were found, so only descriptive statistics could be performed. Third, it cannot be excluded that diagnoses may have been missed in the diagnostic work-up.

In conclusion, we found a low incidence of pathology in children referred for tall stature to a general paediatric clinic, and adult height reduction was seldom indicated. We suggest that the diagnostic work-up and follow-up can be minimal in most children, and propose a diagnostic algorithm for clinical practice.

## Figures and Tables

**Table 1:**
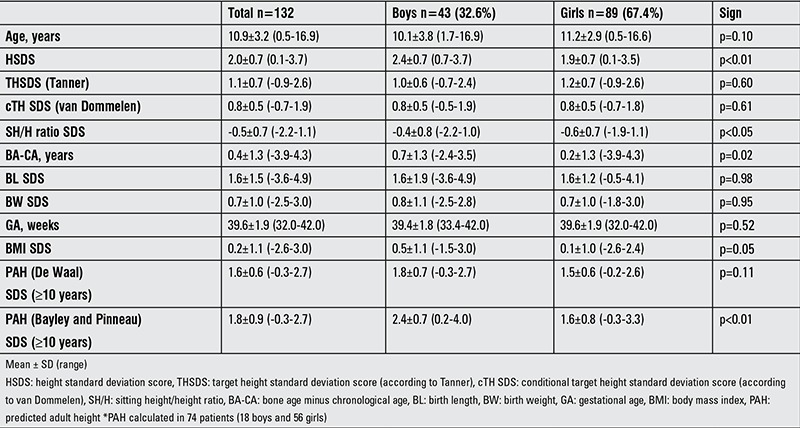
Characteristics of the study population, according to gender

**Table 2:**
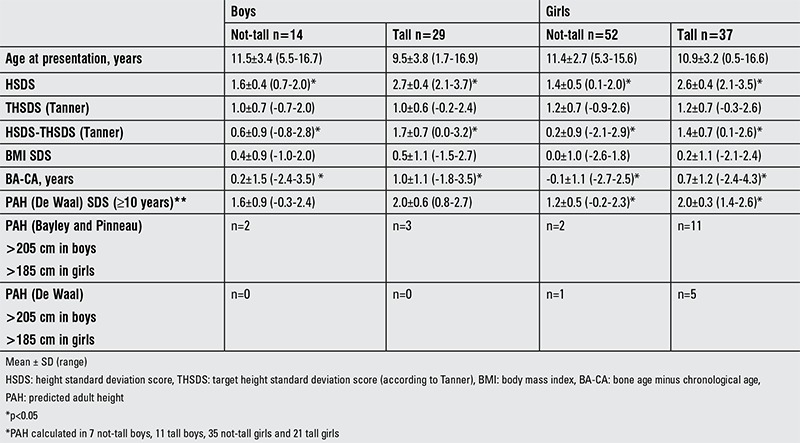
Characteristics of not-tall and tall boys and girls

**Table 3:**
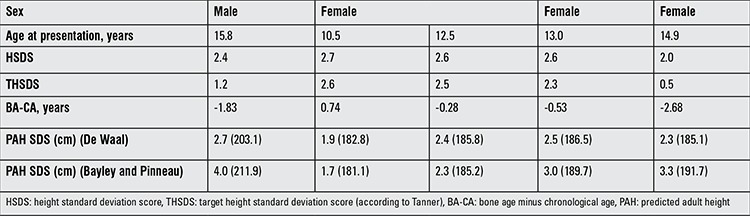
Characteristics of 4 patients who underwent epiphysiodesis
